# The decreasing prevalence of ADHD across the adult lifespan confirmed

**DOI:** 10.7189/jogh.12.03024

**Published:** 2022-06-15

**Authors:** Melissa Vos, Catharina A Hartman

**Affiliations:** University of Groningen, University Medical Center Groningen, Department of Psychiatry, Interdisciplinary Center Psychopathology and Emotion Regulation (ICPE), Groningen, the Netherlands

Attention-Deficit/Hyperactivity Disorder (ADHD) is a neurodevelopmental disorder common in both children and adults. Diagnostic criteria are based on two core symptom domains: inattention and hyperactivity-impulsivity. Additionally, symptoms should first occur before age twelve and be accompanied by impairments in daily life [1]. In childhood, ADHD has an estimated prevalence of 5%-7% [2,3]. It is well known that, during adolescence, inattention symptoms, and in particular hyperactivity-impulsivity symptoms, become milder for a subset of children diagnosed with ADHD. Accordingly, prevalence estimates in adulthood are lower, at 3%-5% [2,4]. It is important to note, however, that compared to childhood ADHD, adult ADHD has received far less research attention, with adult ADHD definitions strongly varying between studies and few large population-based data sets spanning the full adult lifespan available [4-7]. Consequently, it is unclear whether the prevalence of adult ADHD changes with age. That very few older individuals with ADHD are diagnosed and treated highlights the importance of this issue. A recent meta-analysis focusing on individuals with ADHD aged fifty years and older identified a prevalence of 0.23% diagnosed with ADHD and 0.09% treated for ADHD [6]. All things considered, this raises the question whether the prevalence of ADHD is indeed very low beyond age fifty, indicating a decline across adulthood, or whether we underrecognize ADHD in old age.

A recent systematic review and meta-analysis by Song et al. [8] in this journal estimated the global prevalence of adult ADHD across the adult lifespan. The authors had the difficult task of integrating the findings of 40 different data sets. Due to this heterogeneity, two separate ADHD definitions were used: symptomatic adult ADHD (20 studies) and persistent adult ADHD (19 studies), with one study reporting on both. Symptomatic adult ADHD was defined by the presence of above-the-diagnostic-threshold ADHD symptoms in adulthood. Persistent adult ADHD was likewise defined but, in accordance with the criteria for an ADHD diagnosis in the DSM-V, with the additional requirement of a confirmed onset in childhood. Adjusted for the global demographic structure in 2021, Song et al. [8] estimated an overall prevalence of 6.8% for symptomatic adult ADHD and 2.6% for persistent adult ADHD. Importantly, the authors additionally showed that the prevalence of both symptomatic and persistent adult ADHD decreased across adulthood. The decreasing prevalence was more pronounced in persistent adult ADHD, where the prevalence ranged from 5.1% at 18-24 years of age to 0.8% above 60 years of age. For symptomatic adult ADHD, the estimated prevalence decreased from 9.0% in young adulthood to 4.5% above age 60. Following initial suggestive evidence in this direction, the findings of Song et al. establish that the decrease in the population prevalence of ADHD that sets in during adolescence continues across adulthood [7,8].

**Figure:**
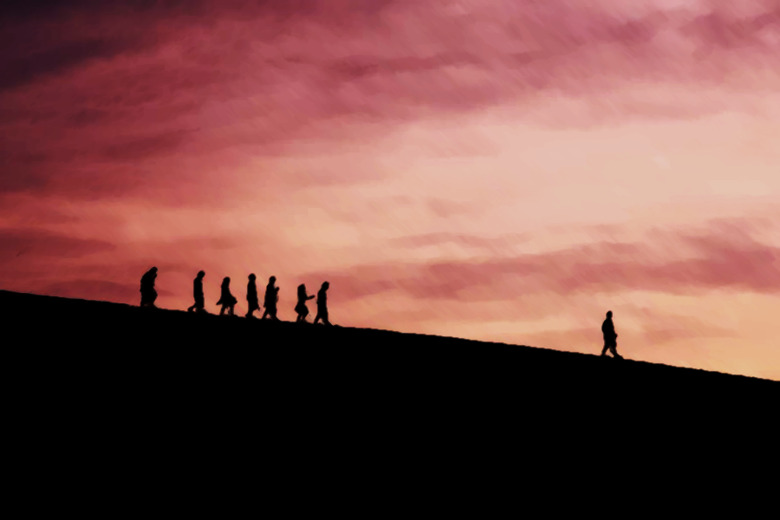
Photo: Artwork by Jehyun Sung, edited to depict the decreasing prevalence of ADHD. Source: https://unsplash.com/photos/6U5AEmQIajg. This photo is free to use under the Unsplash license.

We have recently collected novel data (n = 35 216, collected between 2017 and 2019) in the general population with a partly similar aim in mind to that of the systematic review and meta-analysis by Song et al [8]. This gave us the opportunity to replicate the findings of their meta-analysis, while additionally addressing some of the limitations of the included data sets. We assessed ADHD symptoms with the Dutch version of the adult ADHD DSM-IV questionnaire [9,10]. The questionnaire consists of 11 inattention items and 12 hyperactivity-impulsivity items that are scored from 0 (‘’never or rarely present”) to 3 (‘’very often present”). These 23 items can be used to assess the presence or absence of the 18 DSM-IV ADHD symptoms in the patient’s past six months. A symptom is considered present when one of the corresponding items is rated 2 or higher. The Dutch version of the ADHD DSM-IV questionnaire has shown good psychometric properties [10]. Our study was an add-on study in the Lifelines cohort. Both the original Lifelines Cohort Study and our add-on study are largely representative of the general population in the Northern part of the Netherlands (mean age = 43.0 years, percentage males = 42.6%, and mean highest level of education = 2.21 [ie, middle level on a scale of 1 = low to 3 = high]) and were approved by the ethics committee of the University Medical Centre Groningen [11,12].

Respondents were classified as having symptomatic ADHD when they reported at least five inattention or at least five hyperactivity-impulsivity symptoms [1]. Respondents were classified as having persistent ADHD when they additionally reported an age of onset of their symptoms before age eighteen [1]. The age at onset was retrospectively assessed. We opted for a more lenient interpretation of the age at onset (ie, the DSM-V requires an onset before age twelve) for two reasons; first, the potential difficulties with retrospective reporting of older participants, and second, increasing evidence that ADHD might also have its full clinical onset in adolescence [13,14]. Respondents were classified as having persistent ADHD with impairment when they reported at least five inattention or at least five hyperactivity-impulsivity symptoms, an age at onset before age eighteen, and an impairment level (ie, functional impairment resulting from their ADHD symptoms, a requirement for an ADHD diagnosis) of 6 points or higher on a scale from 1-10 [1]. Note that, while application of the impairment criterion was not explicitly addressed in the meta-analysis by Song et al. and might have differed across the data sets included in the meta-analysis, we did include the assessment of impairment as, in line with DSM, its often an inherent part of a diagnostic instrument.

Song et al. mentioned multiple limitations that may have influenced their findings which the current study can partly address. First, they brought together studies that strongly varied in diagnostic criteria and case definitions, study designs, sampling methods, and study populations in the meta-analysis. Second, they also noted considerable differences between the includes studies in diagnostic tool, DSM version, sex, setting, and investigation period. Most of these limitations evolve around the heterogeneity across studies and reflect the state-of-the-art of the epidemiology of ADHD in adulthood, which lags behind established practices for common adult disorders like Major Depressive Disorder or Substance Use Disorders. While the meta-analysis generalizes across all studies, hopefully sifting out much of the noise in the data, the heterogeneity might nonetheless have impacted the conclusions of the authors. For example, given that studies substantially differed in the age ranges studied, it cannot be distinguished if a declining prevalence rate across adulthood is real or is it the consequence of methodological differences between studies.

We used one study design, study population, diagnostic tool, and investigation period. Moreover, we covered the full adult lifespan between ages 18 and 91. As a single study, our findings obviously do not provide “a global estimate of the prevalence of adult ADHD”, like in Song et al. Rather, our study evades many of the limitations mentioned by Song et al. which are inherent to combining heterogeneous data sets. As such, our findings complement the meta-analytic findings on the prevalence of ADHD across adulthood.

The results of our study are strikingly similar to those reported by Song et al. **Table 1**, which parallels Table 2 in Song et al., shows that prevalence of symptomatic ADHD, persistent ADHD, and persistent ADHD with impairment decreases across adulthood, with an overall prevalence of 8.1% for symptomatic ADHD, 6.5% for persistent ADHD, and 3.4% for persistent ADHD with impairment, the latter most strongly reflecting a DSM-based ADHD diagnosis. (**Table 1**)

**Table 1:** Prevalence of symptomatic ADHD, persistent ADHD, and persistent ADHD with impairment in different parts of the lifespan

	Symptomatic ADHD		Persistent ADHD		Persistent ADHD with impairment	
	**Prevalence**	**Cases**	**Prevalence**	**Cases**	**Prevalence**	**Cases**
**18-24**	15.5	131	15.4	130	8.1	68
**25-29**	13.8	142	12.8	131	7.5	77
**30-34**	14.5	266	12.4	227	6.8	124
**35-39**	12.0	281	10.4	244	5.4	127
**40-44**	10.9	327	9.4	282	5.5	165
**45-49**	9.1	446	7.1	350	3.9	193
**50-54**	7.8	515	5.8	386	3.0	199
**55-59**	6.5	374	4.7	273	2.4	139
**60+**	4.3	379	2.8	246	1.1	100
**Overall (18+)**	8.1	2861	6.5	2269	3.4	1192

ADHD – attention-deficit/hyperactivity disorder

**Figure 1:**
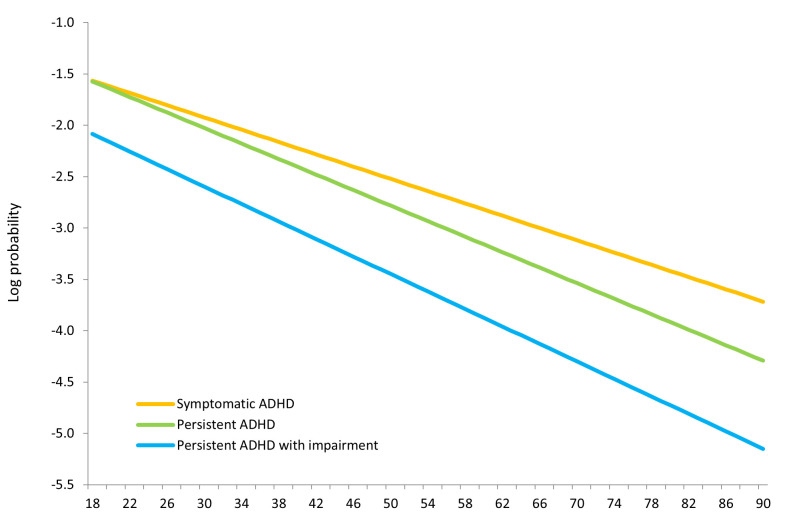
Prevalence of symptomatic ADHD, persistent ADHD, and persistent ADHD with impairment modelled across the adult lifespan using binomial regression with log link. ADHD – attention-deficit/hyperactivity disorder.

To create **Figure 1**, we modelled the prevalence of ADHD across the adult lifespan using binomial regression with a log link function (PR_age_ = 0.96-0.97, 95% CI = 0.95-0.97, *P* < 0.001). This figure clearly illustrates the decreasing prevalence of adult ADHD shown in **Table 1**, and additionally demonstrates that the decrease is stronger for persistent ADHD compared to symptomatic ADHD, corroborating the findings in Figure 2 of Song et al. The non-parallel line of the persistent ADHD group compared to symptomatic ADHD further suggests that adding age of onset decreased the prevalence of adult ADHD by removing older participants with cognitive aging problems, although recall problems on symptoms before age 18 may also have played a role. The almost parallel lines of the persistent ADHD and persistent ADHD with impairment groups indicate that adding impairment as an additional criterium evenly decreases the prevalence of ADHD across adulthood. Thus, illustrating the importance of impairment as an additional diagnostic criterion in prevalence estimation. Our estimate in individuals aged sixty years and older was 1.1%, which is similar to the 0.8% for this age range reported in Song et al. (**Figure 1**).

In sum, based on an independent large sample from the general population, the present study showed strikingly similar results compared to the findings of Song et al. and thus confirmed that ADHD in adults declines across the adult lifespan [8]. In closing, we want to emphasize that while Song et al.’s meta-analysis and our study have shown that the prevalence declines, ADHD is severely underrecognized and undertreated in old age: the lower prevalence in old age identified in the general population is much higher than the prevalence of referred older-aged patients diagnosed with ADHD, which is in turn higher than the prevalence of those treated [6].
